# Phase I study on the safety, pharmacokinetic profile, and efficacy of the combination of TSU-68, an oral antiangiogenic agent, and S-1 in patients with advanced hepatocellular carcinoma

**DOI:** 10.1007/s10637-014-0109-2

**Published:** 2014-05-15

**Authors:** Masafumi Ikeda, Shuichiro Shiina, Kohei Nakachi, Shuichi Mitsunaga, Satoshi Shimizu, Yasushi Kojima, Hideki Ueno, Chigusa Morizane, Shunsuke Kondo, Yasunari Sakamoto, Yoshinari Asaoka, Ryosuke Tateishi, Kazuhiko Koike, Hitoshi Arioka, Takuji Okusaka

**Affiliations:** 1 Department of Hepatobiliary and Pancreatic Oncology, National Cancer Center Hospital East, 6-5-1 Kashiwanoha, Kashiwa, 277-8577 Japan; 2 Department of Gastroenterology, Graduate School of Medicine, Juntendo University, Tokyo, Japan; 3 Department of Gastroenterology, National Center for Global Health and Medicine, Tokyo, Japan; 4 Department of Hepatobiliary and Pancreatic Oncology, National Cancer Center Hospital, Tokyo, Japan; 5 Department of Gastroenterology, Graduate School of Medicine, The University of Tokyo, Tokyo, Japan; 6 Department of Medical Oncology, Yokohama Rosai Hospital, Yokohama, Japan

**Keywords:** Hepatocellular carcinoma, TSU-68, S-1, Chemotherapy, VCAM-1

## Abstract

*Purpose* We aimed to investigate the recommended dose for the combination of TSU-68, a multiple-receptor tyrosine kinase inhibitor targeting vascular endothelial growth factor receptor-2 and platelet-derived growth factor receptor-β, and S-1, an oral fluoropyrimidine, in patients with advanced hepatocellular carcinoma (HCC) based on its associated dose-limiting toxicity (DLT) frequency. We also determined the safety, tolerability, pharmacokinetics (PK), and efficacy of the combination treatment. *Patients and methods* Patients without any prior systemic therapy received 400 mg/day TSU-68 orally and 80 mg/day (level 1) or 100 mg/day (level 2) S-1 for 4 or 2 weeks followed by a 2- or 1-week rest period (groups A and B, respectively). According to the treatment, patients progressed from level 1B to level 2A, then level 2B. Safety and response rates were assessed. *Results* Eighteen patients were enrolled. Two patients at levels 1B and 2A but none at level 2B showed DLTs. The common adverse drug reactions were a decrease in hemoglobin levels, hypoalbuminemia, and anorexia, which were mild in severity (grades 1–2). PK data from levels 1B and 2A indicated that the area under the curve for TSU-68 and 5-fluorouracil was unlikely to be affected by the combination treatment. Response rate, disease control rate, median time to progression, and median overall survival were 27.8 %, 61.1 %, 5.3 months, and 12.8 months, respectively. *Conclusion* The recommended dose for advanced HCC should be 400 mg/day TSU-68 and 100 mg/day S-1 for 4 weeks followed by 2-week rest.

## Introduction

Hepatocellular carcinoma (HCC) is one of the most common cancers worldwide [1]. Surgical resection, percutaneous ethanol injection (PEI), or radiofrequency ablation (RFA) is often performed in patients with curable HCC, whereas transcatheter arterial chemoembolization (TACE) is the treatment of choice for patients with incurable HCC. Sorafenib is the standard chemotherapy for HCC [2, 3], but its efficacy is limited. Therefore, the search for novel medical therapies is essential for HCC management.

As HCC is a highly vascular tumor, a number of anti-angiogenic agents have been tested for its treatment. TSU-68 is a small-molecule, orally administered, multiple-receptor tyrosine kinase inhibitor of the vascular endothelial growth factor receptor-2 (VEGFR-2) and platelet-derived growth factor receptor-β (PDGFR-β) [4–8]. TSU-68 demonstrated favorable efficacy and a good safety profile for HCC [9, 10]. A phase III clinical trial of TSU-68 with TACE (ClinicalTrials.gov identifier NCT01465464 ORIENTAL study) is currently underway.

S-1 is an orally administered fluoropyrimidine anticancer drug that combines tegafur (FT), 5-chloro-2,4-dihydroxypyridine (CDHP), and oteracil potassium (Oxo) in a molar concentration ratio of 1:0.4:1 [11]. CDHP is a competitive inhibitor of dihydropyrimidine dehydrogenase (DPD), a 5-fluorouracil (5-FU)-metabolizing enzyme expressed in the liver. Therefore, prolonged effective concentrations of 5-FU in the plasma and tumor tissue can be achieved via successful inhibition of DPD by CDHP. Oxo, a competitive inhibitor of orotate phosphoribosyltransferase, inhibits the phosphorylation of 5-FU in the gastrointestinal tract, thereby reducing serious 5-FU-related gastrointestinal toxicity. Clinically, S-1 has been shown to be effective against various solid tumors. In Japan, the clinical development of chemotherapies for unresectable, advanced, or recurrent gastric cancers has progressed for many years, and many clinical studies have been conducted. S-1 has showed favorable efficacy and a good safety profile for HCC in a phase I/II study [12]. A phase III clinical trial of S-1 for HCC (Japicdoc identifier JapicCTI-090920 S-CUBE trial) is underway.

Many molecular targeted drugs and anticancer agents have been administered as combination therapies to improve efficacy in various types of cancer. In HCC, combination therapies of molecular targeted agents [13], a molecular-targeted agent and cytotoxic chemotherapy [14–18], and a molecular targeted agent and TACE have been developed [19, 20]. However, no combination therapy has been proven to improve survival [21]. TSU-68 and S-1 have been shown to be effective against HCC in a phase I/II study as single agents. Therefore, we investigated the TSU-68 plus S-1 combination.

This study investigated the recommended dose of TSU-68 plus S-1 in HCC based on the frequency of associated dose-limiting toxicity (DLT). We determined safety, tolerability, pharmacokinetics (PK), and efficacy of the TSU-68 plus S-1 combination in patients with advanced HCC. We also evaluated the expression of endothelial cell markers in patients receiving TSU-68 plus S-1 combination therapy.

## Methods

### Eligibility criteria

The following patients were eligible: (1) patients with confirmed HCC according to histological studies or diagnostic imaging; (2) patients classified as having advanced disease if they were not eligible for or showed disease progression after surgery, PEI, RFA, or TACE; (3) patients with a minimum age of 20 years; (4) patients with an Eastern Cooperative Oncology Group performance status scale score of 0 or 1; (5) patients with measurable disease based on the Response Evaluation Criteria in Solid Tumors (RECIST) v1.0; and (6) patients with a white blood cell count of ≥3,000 cells/mm^3^ or neutrophil count of ≥1,500 cells/mm^3^, hemoglobin level of ≥9.0 g/dL, platelet count of ≥7.5 × 10^4^ cells/mm^3^, total bilirubin level of 2.0 mg/dL, aspartate aminotransferase (AST) and alanine aminotransferase (ALT) levels ≤ upper limit of normal × 5, albumin level of ≥3.0 mg/dL, prothrombin time-international normalized ratio of ≤2.0, and creatinine level ≤ upper limit of normal.

Patients were not eligible if they had received surgery, PEI, RFA, TACE, chemotherapy, or radiotherapy within 30 days before the start of this study. Patients were excluded if there was clinical evidence of central nervous system metastasis, severe cardiovascular disorder, hepatic encephalopathy, uncontrollable pleural effusion or ascites, or a serious infection. Patients requiring prophylactic variceal ligation or sclerotherapy were also excluded.

All patients provided written informed consent. The study was approved by the institutional review board of each of the 3 participating hospitals and was performed in accordance with the principles of the Declaration of Helsinki and Good Clinical Practice Guidelines.

### DLTs

The following were considered as DLTs: (1) a hematological adverse drug reaction of grade 4 (grade 3 febrile neutropenia), (2) a non-hematological adverse drug reaction of grade 3 (excluding AST, ALT, alkaline phosphatase, gamma-glutamyl transpeptidase, Na, and K levels), (3) AST and ALT levels of 10 times higher than the upper limit of normal values, (4) an adverse drug reaction requiring discontinuation of TSU-68 treatment for 15 consecutive days during a cycle, (5) an adverse drug reaction requiring discontinuation of S-1 treatment for 8 consecutive days during a cycle, and (6) an adverse drug reaction requiring discontinuation of TSU-68 plus S-1 treatment.

### Study design and treatment

**Fig. 1:**
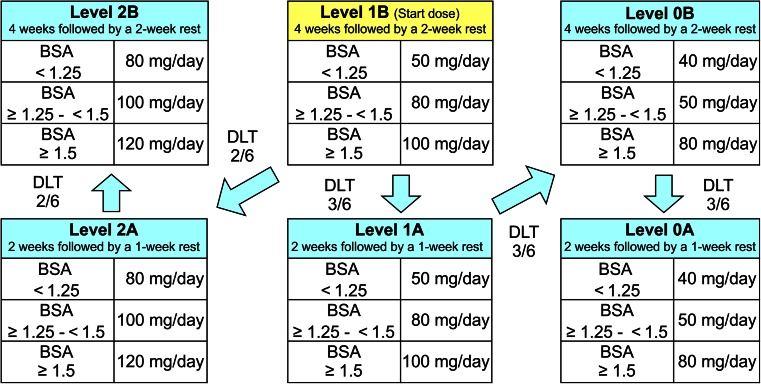
Administration schedule of the TSU-68 plus S-1 combination. Patients received 400 mg/day TSU-68 and 50, 80 or 100 mg/day S-1. The initial dose and treatment duration for S-1 were at level 1B followed by progression to levels 2A–2B in case of dose-limiting toxicity (DLT) ≤ 2/6 or levels 1A-0A in case of DLT ≥ 3/6

The present study was designed as an open-label phase I study. The patients received 400 mg/day TSU-68 orally and one of the following doses of S-1: 50 mg/day (level 0), 80 mg/day (level 1), or 100 mg/day (level 2). TSU-68 and S-1 were administered orally twice daily (after breakfast and supper). For S-1, dose levels were administered according to the following administration and resting schedules: for levels 0B, 1B, and 2B, S-1 was administered continuously for 28 days followed by a 14-day rest period, which completed a treatment cycle, and for levels 0A, 1A, and 2A, a cycle of S-1 was defined as continuous administration of the drug for 14 days followed by a 7-day rest period. Drug administration was continued by repeating the cycle, unless one of the following occurred: progressive disease, unacceptable toxicity, withdrawal of patient consent, or termination of treatment at the attending physician’s discretion. Treatment dose and duration started at level 1B, followed by progression to 2A–2B or 1A–0A in the event of DLTs (Fig. 1). The recommended dose was determined based on the treatment dose, duration, DLTs, toxicity, and efficacy.

### Assessment of efficacy and toxicity

DLTs were recorded for each treatment level, and the rate of DLT development was determined. During each course of treatment, the tumor response was assessed according to RECIST v1.0 by using computed tomography or magnetic resonance imaging, with a maximum slice thickness of 5 mm [22]. Efficacy was evaluated in terms of the response rate (RR), disease control rate (DCR), median time to progression (TTP), and median overall survival (OS) time. TTP was defined as the time from the date of registration to the date when progressive disease was confirmed based on RECIST. OS was defined as the time from the date of registration to the date of death. Physical examination findings and results of serum and urine chemistry analyses were obtained at 1-week intervals for the first cycle and at 2-week intervals during the remaining cycles. Vital signs were assessed as necessary. The severity of all adverse events was evaluated according to the Common Terminology Criteria for Adverse Events, version 3.0.

### PK

We evaluated the PK of co-administered TSU-68 and S-1 on the basis of their plasma concentrations and PK parameters obtained in 12 patients at levels 1B and 2A. Blood samples were collected before dosing and at 1, 2, 3, 4, 6, 9, and 12 h on days 1 and 8 after dosing. TSU-68 plasma concentration was determined using a validated ultraviolet high-performance liquid chromatography method. Plasma concentrations of FT, 5-FU, CDHP, and Oxo were determined using a validated liquid chromatography-tandem mass spectrometry method. PK parameters, including maximum plasma concentration (C_max_), time to reach the maximum plasma concentration, area under curve (AUC), and plasma half-life, were calculated with the non-compartmental method using the PK analysis program WinNonlin Professional (Pharsight Corporation, St. Louis, MO).

### Endothelial cell markers

Blood samples were collected from patients at baseline, and the plasma was isolated and stored at −70 °C until analysis. Concentrations of VEGF-A, VEGF-C, soluble VEGFR-2, vascular cell adhesion molecule 1 (VCAM-1), and E-selectin were quantified using enzyme-linked immunosorbent assay kits (R & D Systems, Minneapolis, MN). All assays were performed in duplicates.

### Statistical analysis

We utilized a full analysis set (FAS), defined as all patients who met the eligibility criteria. All patients were included in the FAS. The rate of DLT, number of patients, incidence rate of all adverse drug reactions, PK, and efficacy were determined. Efficacy was assessed in terms of the RR, DCR, TTP, and OS. The median TTP and OS were assessed using the Kaplan-Meier method, and 95 % confidence intervals (CI) were calculated. In addition, the relationship between TTP and endothelial cell markers at baseline was examined. TTP was assessed at high and low levels of endothelial cell markers. Hazard ratios (HRs) and 95 % CIs were estimated by using the Cox proportional-hazards model. Changes in biomarker concentrations from baseline were evaluated using the exact-paired Wilcoxon test (two-sided). A *p*-value of <0.05 was considered statistically significant.

## Results

### Patient characteristics

Between December 2008 and June 2010, 18 patients (6 each at levels 1B, 2A, and 2B) were enrolled at National Cancer Center East, National Cancer Center, and The University of Tokyo Hospital in Japan. Baseline demographics and disease characteristics are summarized in Table 1.

**Table 1:** Patient characteristics

Variable	Category	Level 1B	Level 2A	Level 2B	Total
Number of patients	Total	6	6	6	18
Sex	Male	5	6	4	15
	Female	1	0	2	3
Age (years)	Median	72.5	74	65.5	71.5
	Range	58-75	64-85	60-73	58-85
ECOG performance status	0	6	4	6	16
	1	0	2	0	2
Viral markers	HBs Ag+	1	2	0	3
	HCV Ab+	3	4	5	12
BCLC stage	Intermediate	1	3	3	7
	Advanced	5	3	3	11
Child-Pugh status	A	6	6	6	18

Abbreviations: ECOG performance status, Eastern Cooperative Oncology Group performance status

HBs Ag, hepatitis B surface antigen; HCV Ab, hepatitis C antibody

BCLC stage, Barcelona-Clinic Liver Cancer staging system

Evaluation of liver function revealed that all 18 patients had Child-Pugh A cirrhosis. Of these, 7 patients (38.9 %) were at an intermediate stage and 11 (61.1 %) were at an advanced stage according to the Barcelona-Clinic Liver Cancer staging system [23, 24]. Seven (38.9 %) patients had previously undergone surgeries and 15 (83.3 %) had previously received local therapy. None of the 18 patients had ever received sorafenib.

### Dose delivery

Eighteen patients (6 each at levels 1B, 2A, and 2B) received 400 mg of TSU-68 orally per day and one of the following doses of S-1: 80 mg/day (level 1) or 100 mg/day (level 2). For levels 1B–2B, the median relative dose intensities of TSU-68 were 70.0 %, 76.9 %, and 87.1 %, respectively, and the corresponding values for S-1 were 70.9 %, 75.4 %, and 87.0 %, respectively. The median duration of TSU-68 treatment was 83.3 days for level 1B, 49.8 days for level 2A, and 225.8 days for level 2B; the corresponding median durations of S-1 treatment were 67.8 days, 36.5 days, and 159.5 days, respectively.

### Safety

Two patients at level 1B exhibited DLTs. The first patient experienced grade 3 gastrointestinal bleeding, grade 3 gastric ulcer, grade 4 hemoglobin, grade 3 nausea, and grade 3 vomiting, while the second patient had grade 2 ascites.

At level 2A, two patients of DLTs were reported, one of grade 3 fatigue and the other of grade 3 hand-foot skin reaction and grade 3 rash. However, no DLTs were observed at level 2B.

Table 2 shows the drug-related adverse reactions reported in at least 40 % of all patients or at ≥ grade 3. The most common adverse events, regardless of grade, were a decreased hemoglobin level (83.3 %), anorexia (77.8 %), hypoalbuminemia (72.2 %), localized edema (66.7 %), fatigue (61.1 %), nausea (61.1 %), a decreased platelet count (61.1 %), urine color change (55.6 %), hyperbilirubinemia (50.0 %), exfoliative rash (50.0 %), and hyperpigmentation (50.0 %). The most common grade 3 or 4 hematological toxic effect was lymphopenia (22.2 %), while the most common non-hematological toxic effects were fatigue (22.2 %), anorexia (16.7 %), and an elevated serum AST level (16.7 %).

**Table 2:** Adverse drug reactions according to grade (n = 18) observed in the study population

	Level 1B		Level 2A		Level 2B		Total	
	pts	Gr ≥3	pts	Gr ≥3	pts	Gr ≥3	pts	Gr ≥3
Hemoglobin decrease	4	2	6	0	5	0	15	2
Anorexia	5	0	5	2	4	1	14	3
Hypoalbuminemia	4	0	4	0	5	0	13	0
Localized edema	4	0	4	0	4	0	12	0
Fatigue	4	1	3	2	4	1	11	4
Nausea	4	1	4	0	3	0	11	1
Platelet count decrease	4	0	5	1	2	0	11	1
Urine color change	4	0	3	0	3	0	10	0
Hyperbilirubinemia	2	1	4	0	3	0	9	1
Exfoliative rash	4	0	3	1	2	0	9	1
Skin hyperpigmentation	2	0	3	0	4	0	9	0
Lymphopenia	3	2	4	2	1	0	8	4
Aspartate aminotransferase level increase	4	2	1	1	3	0	8	3
Neutrophil count decrease	3	0	2	1	3	1	8	2
Hyponatremia	3	2	3	0	1	0	7	2
Vomiting	3	1	2	0	2	0	7	1
Alanine aminotransferase level increase	2	1	2	1	1	0	5	2
Hypokalemia	2	1	1	1	0	0	3	2
Hand-foot skin reaction	0	0	2	1	1	0	3	1
Pleural effusion	1	0	1	1	0	0	2	1
Gastric hemorrhage	1	1	0	0	0	0	1	1
Gastric ulcer	1	1	0	0	0	0	1	1
Liver abscess	0	0	0	0	1	1	1	1
Thrombosis	0	0	1	1	0	0	1	1
Blood glucose decrease	1	1	0	0	0	0	1	1
Myocardial ischemia	0	0	0	0	1	1	1	1
Pericardial effusion	0	0	1	1	0	0	1	1
Cardiac failure	0	0	1	1	0	0	1	1

Results are expressed as the worst TSU-68 or S-1-related adverse event that occurred in at least 40% of patients or were classified as grade ≥3

All adverse events are documented according to the National Cancer Institute Common

Toxicity Criteria version 3.0

Abbreviations: pts, number of patients; Gr ≥3, grade ≥3

One patient treated at level 2A died of heart failure. Three patients at level 1B and 2A and 1 patient at level 2B were hospitalized because of serious adverse events. Treatment was discontinued in 3 patients because of an adverse reaction of ≥ grade 3, including grade 4 pericardial effusion/grade 4 pleural effusion, grade 3 fatigue, and grade 3 hand-foot reactions/grade 3 rash.

### PK

**Fig. 2:**
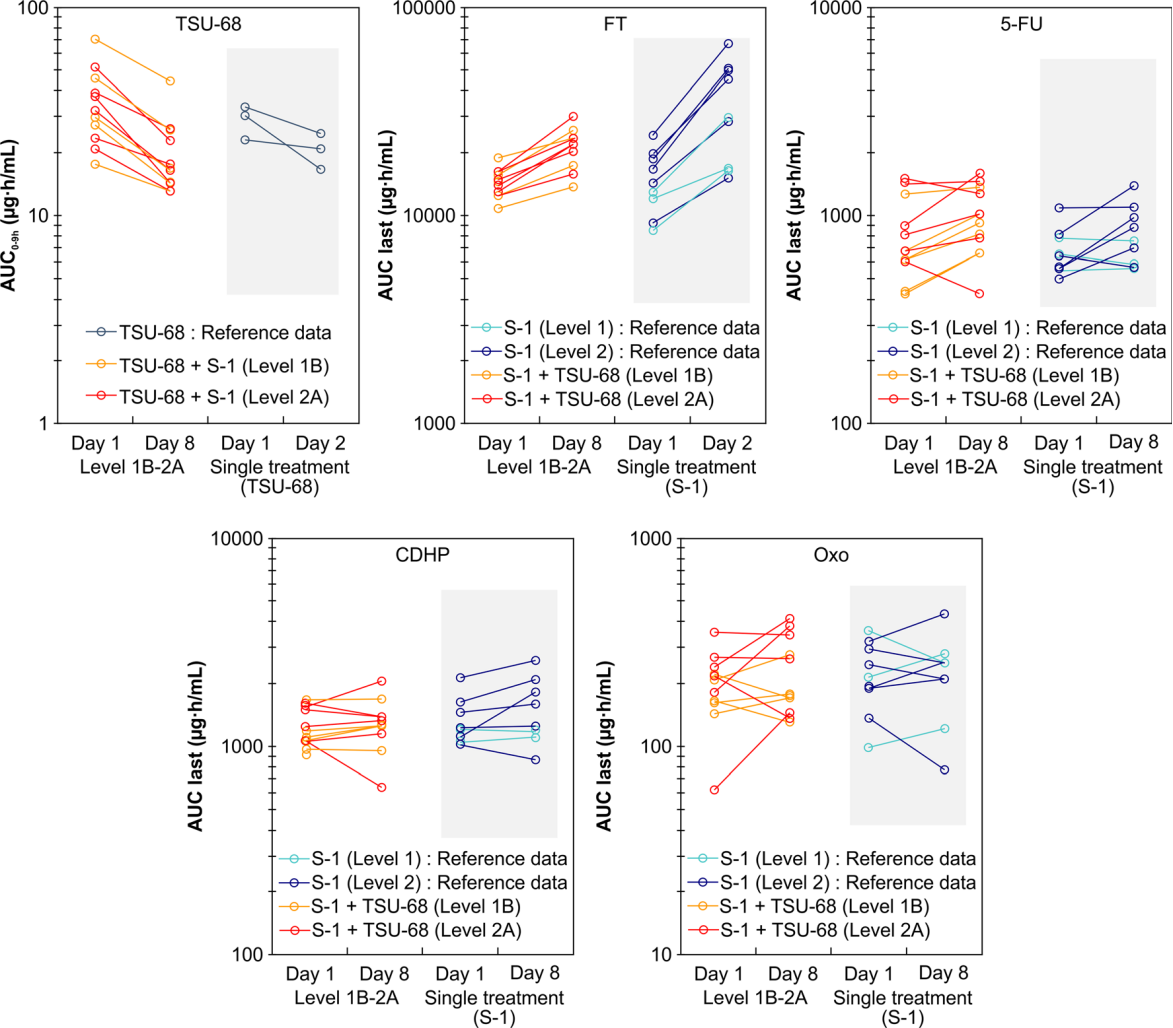
Pharmacokinetics analysis of data from 12 patients at levels 1B and 2A. The results indicated that the areas under the curve (AUC) of TSU-68 and 5-FU were unlikely to be affected by the combined administration of TSU-68 plus S-1. The AUC of FT appeared to decrease on the administration of TSU-68 plus S-1 compared to that on S-1 alone. However, other components were not affected by the administration of TSU-68 plus S-1 in combination compared to that of S-1 alone

The PK parameters of TSU-68 after co-administration with S-1 were the same for level 1B and 2A, as shown in Fig. 2. In addition, these PK parameters were similar to those for TSU-68 administered alone.

The PK parameters of S-1 components and 5-FU at level 2A were slightly higher than the corresponding values at level 1B, as expected after the dose increase. The C_max_ and AUC of 5-FU, CDHP, and Oxo were not markedly different between day 1 and day 8, while those of FT were higher on day 8 than on day 1 because of accumulated exposure with repeated administration of S-1.

### Efficacy

The anti-tumor effect of TSU-68 and S-1 was evaluated in the 18 patients included in the FAS (Table 3). At level 1B, 1 of the 6 patients showed partial response (PR), corresponding to a response rate of 16.7 %. At level 2B, 4 patients had PR, corresponding to a response rate of 66.7 %.

**Table 3:** Efficacy results in all patients

	Level 1B	Level 2A	Level 2B
	(n = 6)	(n = 6)	(n = 6)
Complete response, n (%)	0 (0.0)	0 (0.0)	0 (0.0)
Partial response, n (%)	1 (16.7)	0 (0.0)	4 (66.7)
Stable disease, n (%)	2 (33.3)	3 (50.0)	1 (16.7)
Progressive disease, n (%)	3 (50.0)	3 (50.0)	1 (16.7)
Not evaluated, n (%)	0 (0.0)	0 (0.0)	0 (0.0)
Response rate, n (%)	1 (16.7)	0 (0.0)	4 (66.7)
95 % CI (%)	0.4–64.1	0.0–45.9	22.3–95.7
Disease control rate, n (%)	3 (50.0)	3 (50.0)	5 (83.3)
95 % CI (%)	11.8–88.2	11.8–88.2	35.9–99.6
Median TTP (month)	3.9	2.4	8
95 % CI (month)	2.5–6.3	1.9– -	5.3–12.2
MST (month)	10.7	14.9	16.3
95 % CI (month)	5.7– -	11.3–20.8	11.6– -

Abbreviations: n, number; TTP, time to progression; MST, median survival time

**Fig. 3:**
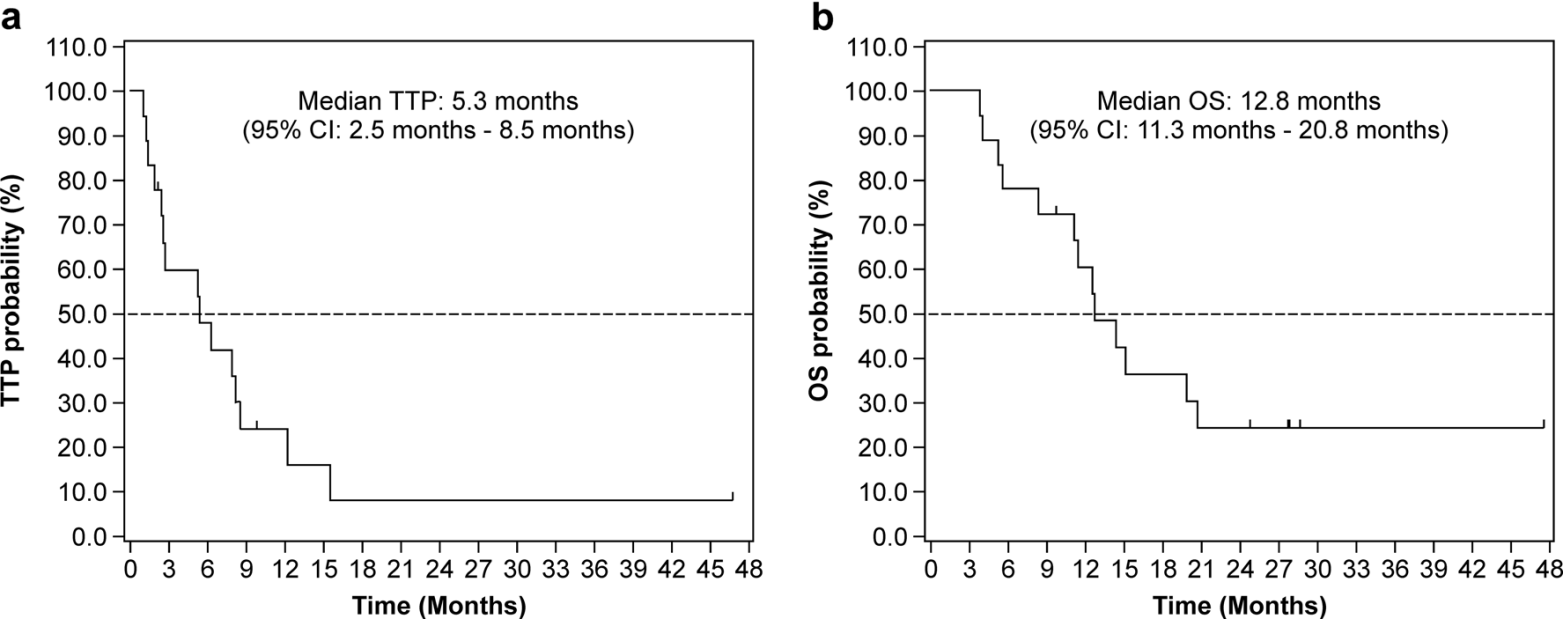
Kaplan-Meier analysis demonstrating the time to progression (a) and overall survival (b) of advanced hepatocellular carcinoma patients treated with the TSU-68 plus S-1 combination

The median TTP was 3.9 months at level 1B, 2.4 months at level 2A, 8.0 months at level 2B, and 5.3 months for all patients (Fig. 3). OS was evaluated for 18 patients in the FAS and was 10.7 months at level 1B, 14.9 months at level 2A, 16.3 months at level 2B, and 12.8 months for all patients (Fig. 3).

### Endothelial cell markers

Biomarker analysis showed that TTP was significantly longer in patients with low VCAM-1 levels than that in other patients (hazard ratio, 0.26; 95 % CI, 0.08–0.85; *p* = 0.018) (Table 4). No significant difference was observed with other endothelial cell markers examined.

**Table 4:** Biomarker analysis

	Median	< Median conc.	≥ Median conc.	Hazard ratio	95 % CI	*p* value^*^
		Median TTP (days)	Median TTP (days)			
VCAM-1	1,310	372	77	3.89	1.17–12.9	0.018
ELAM-1	52	193	157	1.14	0.41–3.18	0.799
PDGF-BB	1793.1	79	239	0.81	0.28–2.37	0.704
Total PAI-1	15	193	157	1.99	0.68–5.84	0.205
FGF acidic	31.3	-	160	-	-	-
bFGF	10	-	160	-	-	-
PDGF-AA	418.55	157	239	1.09	0.39–3.03	0.865
VEGF	51.5	157	239	0.53	0.18–1.6	0.255
sVEGFR-2	7170.35	160	193	1.01	0.36–2.8	0.992
VEGF-C	1092.1	79	239	0.76	0.27–2.1	0.594
VEGFR-3	8063.45	193	160	2.1	0.67–6.56	0.192

*Log-rank test

Abbreviations: conc, concentration; TTP, time to progression

VCAM-1, vascular cell adhesion molecule 1

ELAM-1, endothelial-leukocyte adhesion molecule 1

PDGF-BB, platelet-derived growth factor BB

PAI-1, plasminogen activator inhibitor 1

FGF, fibroblast growth factor

bFGF, basic fibroblast growth factor

PDGF-AA, platelet-derived growth factor AA

VEGF, vascular endothelial growth factor

sVEGFR-2, soluble vascular endothelial growth factor receptor 2

VEGF-C, vascular endothelial growth factor C

VEGFR-3, vascular endothelial growth factor receptor 3

## Discussion

In the present study, we determined the recommended dose of the TSU-68 plus S-1 combination in patients with advanced HCC based on the frequency of associated DLTs. We also investigated the safety, tolerability, PK, and efficacy of the TSU-68 plus S-1 combination in our study population. For combination therapy, it is important to achieve efficacy without worsening of PK parameters or adverse drug reactions. Investigation of molecular markers in TSU-68 plus S-1 combination therapy merits additional research.

We determined the safety of several treatment levels by examining the frequency of associated DLTs. Such events were observed in 2 patients at level 1B (1 with gastrointestinal bleeding, gastric ulcer, hemoglobin, nausea, and vomiting, and the other 1 with ascites) and in 2 patients at level 2A (1 with fatigue and the other 1 with hand-foot skin reaction and rash). However, at level 2B, none of the patients demonstrated DLTs. With regard to adverse drug reactions of grade 3 or higher severity, 17 events were noted at levels 1B and 2A and 5 events were noted at level 2B. Overall, treatment at level 2B resulted in the least severe DLTs, less serious adverse events, and fewer adverse reactions of grade 3 or higher severity.

When we compared the adverse drug reaction incidence in this trial with those reported in independent trials for TSU-68 [9] and S-1 [12], in which each agent was administered alone to patients with HCC, we found that TSU-68 plus S-1 combination therapy did not increase adverse drug reactions. Compared to the trial in which TSU-68 was administered alone, the incidences of anorexia, localized edema, fatigue, nausea, skin pigmentation, hemoglobin level decrease, hypoalbuminemia, thrombopenia, and hyperbilirubinemia were higher in the present study by more than 20 %. However, compared to the trial in which S-1 was administered alone, the incidences of localized edema and nausea in the present study were higher by more than 20 % while those of some other adverse drug reactions were lower by more than 20 %. Nevertheless, there was no difference in the adverse drug reaction rate between the TSU-68 and S-1 combination therapy and TSU-68 or S-1 administered alone. The common adverse drug reactions of the TSU-68 plus S-1 combination were mild in severity (grades 1–2). Our results demonstrated that the TSU-68 plus S-1 combination was well tolerated in patients with HCC.

We compared the PK data on TSU-68 plus S-1 combination therapy with the PK data on S-1 or TSU-68 alone [9, 12]. Our results suggested that TSU-68 PK parameters were unlikely to be affected by co-administration with S-1. The C_max_ and AUC of TSU-68 on day 8 were lower than those on day 1 for cytochrome P450 1A2, as has been reported previously [9]. For S-1, exposure to FT after repeated co-administration with TSU-68 tended to be lower than that reported previously for S-1 administered alone [12]. However, there were no apparent differences in the PK parameters of 5-FU, CDHP, and Oxo between the two studies. Our data indicated that the PK parameters of TSU-68 and S-1 were independent and hence, unaffected by combined administration, with the exception of exposure to FT.

Next, we compared the effectiveness of TSU-68 and S-1 administered alone and in combination. TTP and OS for the 18 patients receiving TSU-68 plus S-1 combination therapy were 5.3 months and 12.8 months, and 8.0 months and 16.3 months at level 2B, respectively. On the other hand, the progression-free survival (PFS) and OS for patients receiving S-1 alone were 3.7 months and 16.6 months, respectively, and the TTP and OS for patients with TSU-68 alone were 2.1 months and 13.1 months. Therefore, the efficacy of the combination therapy at level 2B treatment was better than that of either TSU-68 or S-1 treatment alone.

We also compared the efficacy of the TSU-68 plus S-1 combination administered at level 2B in this study to that of sorafenib plus S-1 [14] and sorafenib plus Dox [15]. The PFS was 3.9 months and 6.0 months for sorafenib plus S-1 and sorafenib plus Dox, respectively, while the corresponding OS was 10.4 months and 13.7 months, respectively. Therefore, the efficacy of level 2B treatment was better than that of the other two reported combinations.

Taken together, our results indicate that the TSU-68 plus S-1 combination therapy is safe and efficacious; nevertheless, further investigation of the treatment at level 2B, in particular, is warranted.

Furthermore, we evaluated expression of endothelial cell markers in patients receiving the TSU-68 plus S-1 combination therapy. VCAM-1 is aberrantly expressed in breast cancer cells and mediates pro-metastatic tumor-stroma interactions [25, 26]. In HCC, serum VCAM-1 level appears to reflect the severity of the underlying chronic liver disease rather than the tumor status [27, 28], and low preoperative serum VCAM-1 levels are predictive of better disease-free survival after surgery [28]. Our results suggest that the VCAM-1 level may be used as a predictive factor for TSU-68 plus S-1 combination therapy. However, these data are preliminary and further research will be needed to confirm the relationship between VCAM-1 and prognosis in TSU-68 plus S-1 combination therapy.

In conclusion, our findings reveal that the TSU-68 plus S-1 recommended dose for advanced HCC is 400 mg/day TSU-68 and 100 mg/day S-1 for 4 weeks followed by 2-week rest. TSU-68 plus S-1 combination was well tolerated and had favorable efficacy in patients with advanced HCC. Biomarker analysis showed that VCAM-1 may be a possible predictive marker for response. Further study is necessary to confirm whether TSU-68 plus S-1 combination therapy is a therapeutic option for advanced HCC and if VCAM-1 is a possible predictive marker for response.
